# Complete mitochondrial genome of the praying mantis *Arria pallida* (Zhang, 1987) (Mantodea: Haanidae)

**DOI:** 10.1080/23802359.2020.1820400

**Published:** 2020-09-18

**Authors:** Yingjian Wang, Yongqiang Wang, Jiankun Long, Lin Yang, Xiangsheng Chen

**Affiliations:** a Institute of Entomology, Guizhou University, Guiyang, P. R. China; b The Provincial Special Key Laboratory for Development and Utilization of Insect Resources of Guizhou, Guizhou University, Guiyang, P. R. China; c Guizhou Key Laboratory for Plant Pest Management of Mountainous Region, Guizhou University, Guiyang, P. R. China; d College of Animal Sciences, Guizhou University, Guiyang, P. R. China

**Keywords:** Mitochondrial genome, praying mantis, Arrini, phylogeny

## Abstract

The complete mitochondrial genome of *Arria pallida* is 16,240 bp in length, containing 13 protein-coding genes (PCGs), 2 ribosomal RNA (rRNAs), a control region (D-loop), and 22 transfer RNA (tRNAs). The phylogenetic analysis included 18 species within 10 families of Mantodea using maximum likelihood (ML) method. The result showed that *A. pallida* is sister to *Haania* sp., both of which form a sister clade with *Caliris* sp.

Mantodea (praying mantis) is a group of camouflaged and predatory insects, encompassing about 2500 species (Wieland and Svenson 2018). *Arria pallida* (Zhang 1987) belongs to Arrini within Haaniidae. Arrini is a clade of oriental praying mantis (Schwarz and Roy 2018, 2019) and it is a rare tribe with few reports. Herein, the first complete mitochondrial genome of *Arria pallida* and its phylogenetic relationship with other Mantises is provided. This represents the first publish genomic data of Arrini.

The *A. pallida* sample was collected from Fanjingshan Nature Reserve (108.65°N, 27.91°E), Guizhou province, southwest of China, during August 2018. The specimen was stored in absolute ethanol and kept in the Institute of Entomology, Guizhou University (voucher no. Ha.A-201808fj01), Guiyang, China (IEGU). Sequencing was conducted using the Illumina HiSeq platform (San Diego, CA, USA). All the genes were predicted using MitoZ (Meng et al. 2019).

The complete mitochondrial genome of *A. pallida* (GenBank accession MT594484, link: https://www.ncbi.nlm.nih.gov/nuccore/MT594484) is 16,240 bp in length, with base composition: 40.2% A, 38.0% T, 13.1% C, and 8.7% G. Containing 13 PCGs, two *rRNA* genes, a putative control region (D-loop), and 22 *tRNA* genes. The N-strand codes nine PCGs (*nd2, co1, co2, atp8, atp6, co3, nd3, nd6,* and *cytb*), while the J-strand codes the remaining four PCGs (nd5, nd4, nd4l, and nd1). The gene arrangement of *A. pallida* is similar to the common type of putative ancestor of insects. Five start codons of PCGs exist: ATG (*nad2, cox2, atp6, cox3, nad5, nad4, nad4l,* and *cytb*), TTG (*cox1*), ATC (*nad3*), ATT (*nad6*), ATA (*atp8* and *nad1*). Most of the 13 PCGs had TAA as stop codon whereas cox2, cox3 had T and nad5 had TA. The rrnL is 1320 bp (81.3% AT) and the rrnS is 786 bp (79.4% AT).

Phylogenetic analysis (Figure 1) was constructed using ML method with 13 PCG sequences extracted from 18 species within 10 families using IQtree version 1.6.12 (Nguyen et al. 2015). Metallyticidae was set as outgroup. *Arria pallida* was found to cluster with *Haania* sp., both of which form a sister clade with Caliris sp. This finding is consistent with the classification system set up by Schwarz and Roy (2019).

**Figure 1.**
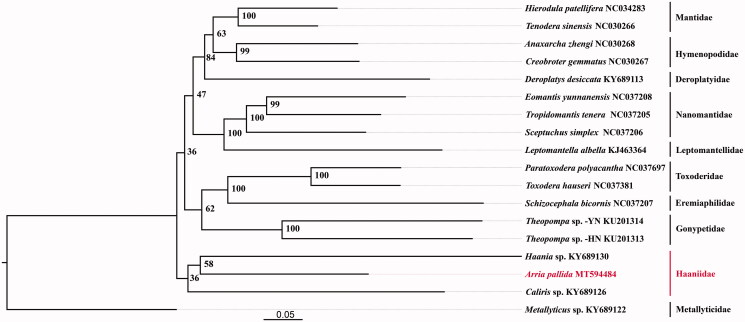
Phylogenetic tree showing *Arria pallida* relationship with other Mantodea, based on 13 protein-coding genes.
